# Supplementary material for: Genome-Wide Identification and Expression Analysis of nsLTP Gene Family in Rapeseed (Brassica napus) Reveals Their Critical Roles in Biotic and Abiotic Stress Responses
Source: Int J Mol Sci. 2022 Jul 28;23(15):8372. doi: 10.3390/ijms23158372 (PMC9368849; doi:10.3390/ijms23158372)
Supplement: Supplementary file 1 [file ijms-23-08372-s001.zip › ijms-1829603-supplementary/Captions for supplementary materials-BnLTP 20220716.pdf]

Genome-wide identification and expression analysis of *nsLTP* gene family in rapeseed (*Brassica napus*) reveals their critical roles in biotic and abiotic stress responses

Yufei Xue, Chunyu Zhang, Rui Shan, Xiaorong Li, Alain Tseke Inkabanga, Lejing Li, Huanhuan Jiang and Yourong Chai*

Caption for supplementary materials:

Table S1. Basic information of *BnLTP* family genes identified in *Brassica napus* and their encoding proteins

Table S2. Basic information of *BrLTP* family genes identified in *Brassica rapa* and their encoding proteins

Table S3. Basic information of *BoLTP* family genes identified in *Brassica oleracea* and their encoding proteins

Table S4. Basic information of *AtLTP* family genes identified in *Arabidopsis thaliana* and their encoding proteins

Table S5. The sequences of eight cysteine motifs (8CMs) of nsLTP proteins from *B. napus*, *B. rapa*, *B. oleracea* and *A. thaliana*

Table S6. Basic information of *BnLTP* gene clusters

Table S7. Duplication patterns of *BnLTP* family genes identified in *B. napus*

Table S8. Promoter sequences of *BnLTP* family genes identified in *B. napus*

Table S9. *cis*-Elements identified in *BnLTP* family gene promoters

Table S10. SSR loci identified in *BnLTP* family genes or their promoters

Table S11. The 42 samples of *B. napus* cultivar ZS11 used in RNA-seq expression analysis

Table S12. Average FPKM values of three biological duplicates for the expression of *BnLTP* genes in 42 various tissues and organs.

Table S13. The FPKM values of *BnLTP* genes expressed in eight LMD-acquired tissues from the globular seed. EP, embryo proper; MCE, micropylar endosperm; PEN, peripheral endosperm; CZE, chalazal endosperm; CPT, chalazal proliferating tissue; CZSC, chalazal seed coat; ISC, inner seed coat; OSC, outer seed coat.

Table S14. The FPKM values of *BnLTP* genes expressed in three LMD-acquired funiculus tissues and the whole funiculus in *B. napus.* Cor, cortex; Epi, Epidermis; Vasc, vasculature; WF, whole funiculus.

Table S15. Average FPKM values of three biological duplicates for the response of *BnLTP* family genes to *S. sclerotiorum*

Table S16. Average FPKM values of two or three biological duplicates for the response of *BnLTP* family genes to *L. maculans*

Table S17. The average FPKM values of the expression levels of *BnLTP* family genes under multiple abiotic stresses

Table S18. The average FPKM values of the expression levels of *BnLTP* family genes under heat and drought

Table S19. The average FPKM values of expression levels of *BnLTP* family genes under cold and freezing treatments

Table S20. The FPKM values of the expression levels of *BnLTP* family genes under IAA treatment (WTZIAA) and control (WTFIAA)

Table S21. The TPM values of the expression levels of *BnLTP* family genes in *B. napus* seed germination

Table S22. The RPKM values of *BnLTP* family genes expressed in brown seeds and yellow mutation

Table S23. The RNA-seq data of *BnLTP* family DEGs expressed in 20 DAP seeds of transgenic rapeseeds (Westar cultivar) overexpressing *pBAN::BnTT1*

Table S24. The RNA-seq data of *BnLTP* family DEGs expressed in 20 DAP seeds of transgenic rapeseeds (Westar cultivar) overexpressing *pNapA::BnTT1*

Table S25. The RNA-seq data of *BnLTP* family DEGs expressed in 25 DAP seeds of transgenic rapeseeds (Westar cultivar) overexpressing *pNapA::BnbZIP67*

Table S26. Primers for the qRT-PCR used in this study.

Figure S1. Phylogenetic analysis, exon-intron structure and MEME motifs of *BnLTP* family genes

Figure S2. Chromosome distribution of *BnLTP* family genes

Figure S3. The heatmap (containing gene names) of expression patterns of *BnLTP* genes in 42 various tissues and organs

Figure S4. Expression patterns of 38 *BnLTP* gene clusters in 42 various tissues and organs

Figure S5. Expression patterns of 74 *BnLTP* duplicated gene groups in 42 various tissues and organs

Figure S6. Expression patterns of *BnLTP* genes in eight LMD-acquired tissues from the globular seed.. EP, embryo proper; MCE, micropylar endosperm; PEN, peripheral endosperm; CZE, chalazal endosperm; CPT, chalazal proliferating tissue; CZSC, chalazal seed coat; ISC, inner seed coat; OSC, outer seed coat.

Figure S7. Expression patterns of *BnLTP* family genes in three LMD-acquired tissues from the globular-stage funiculus. Expressions were identified to be tissue-specific if they were significantly differentially expressed at a level at least 4-fold higher in one tissue relative to the other tissues. Heatmap showed the tissue-specificity of transcripts. Cor, cortex; Epi, Epidermis; Vasc, vasculature.

Figure S8. The heatmap of expression levels of *BnLTP* genes under cold and freezing treatments

Figure S9. The change dynamics of numbers of expressed (**a**) and responsive (**b**) *BnLTP* genes in *B. napus* seed germination
