# Supplementary material for: Genome-Wide Identification and Expression Analysis of nsLTP Gene Family in Rapeseed (Brassica napus) Reveals Their Critical Roles in Biotic and Abiotic Stress Responses
Source: Int J Mol Sci. 2022 Jul 28;23(15):8372. doi: 10.3390/ijms23158372 (PMC9368849; doi:10.3390/ijms23158372)
Supplement: Supplementary file 1 [file ijms-23-08372-s001.zip › ijms-1829603-supplementary/Figure S9.pdf]

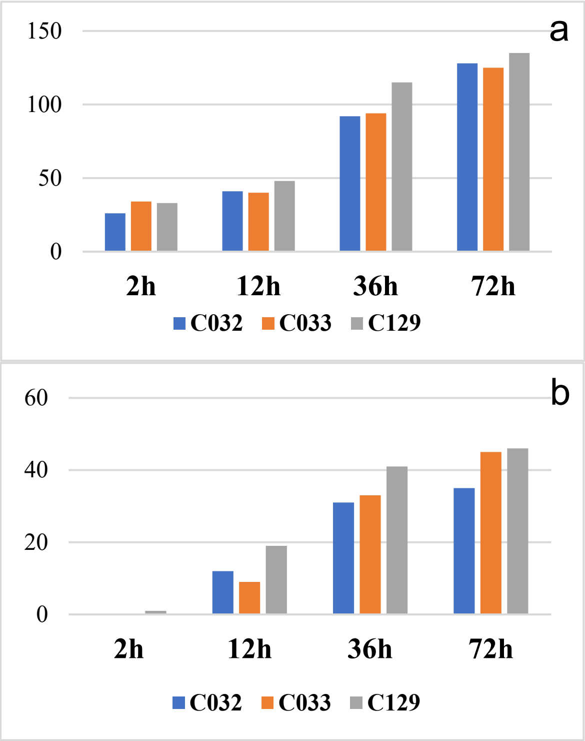

Figure S9. The change dynamics of numbers of expressed (**a**) and responsive (**b**) *BnLTP* genes in *B. napus* seed germination
