# Supplementary figures and images for: Genome-Wide Identification and Expression Analysis of nsLTP Gene Family in Rapeseed (Brassica napus) Reveals Their Critical Roles in Biotic and Abiotic Stress Responses
Source: Int J Mol Sci. 2022 Jul 28;23(15):8372. doi: 10.3390/ijms23158372 (PMC9368849; doi:10.3390/ijms23158372)

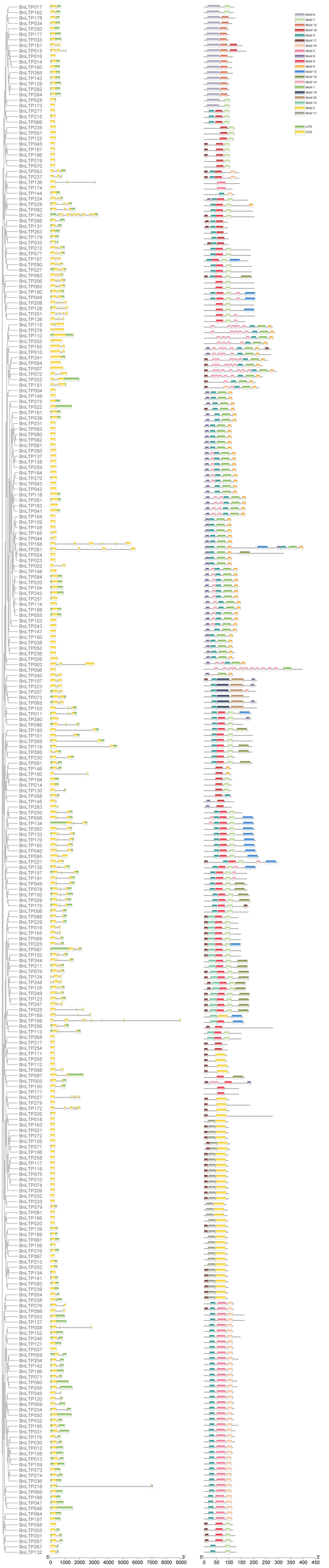

Supplement: Supplementary file 1 [file ijms-23-08372-s001.zip › ijms-1829603-supplementary/Figure S1.tif]

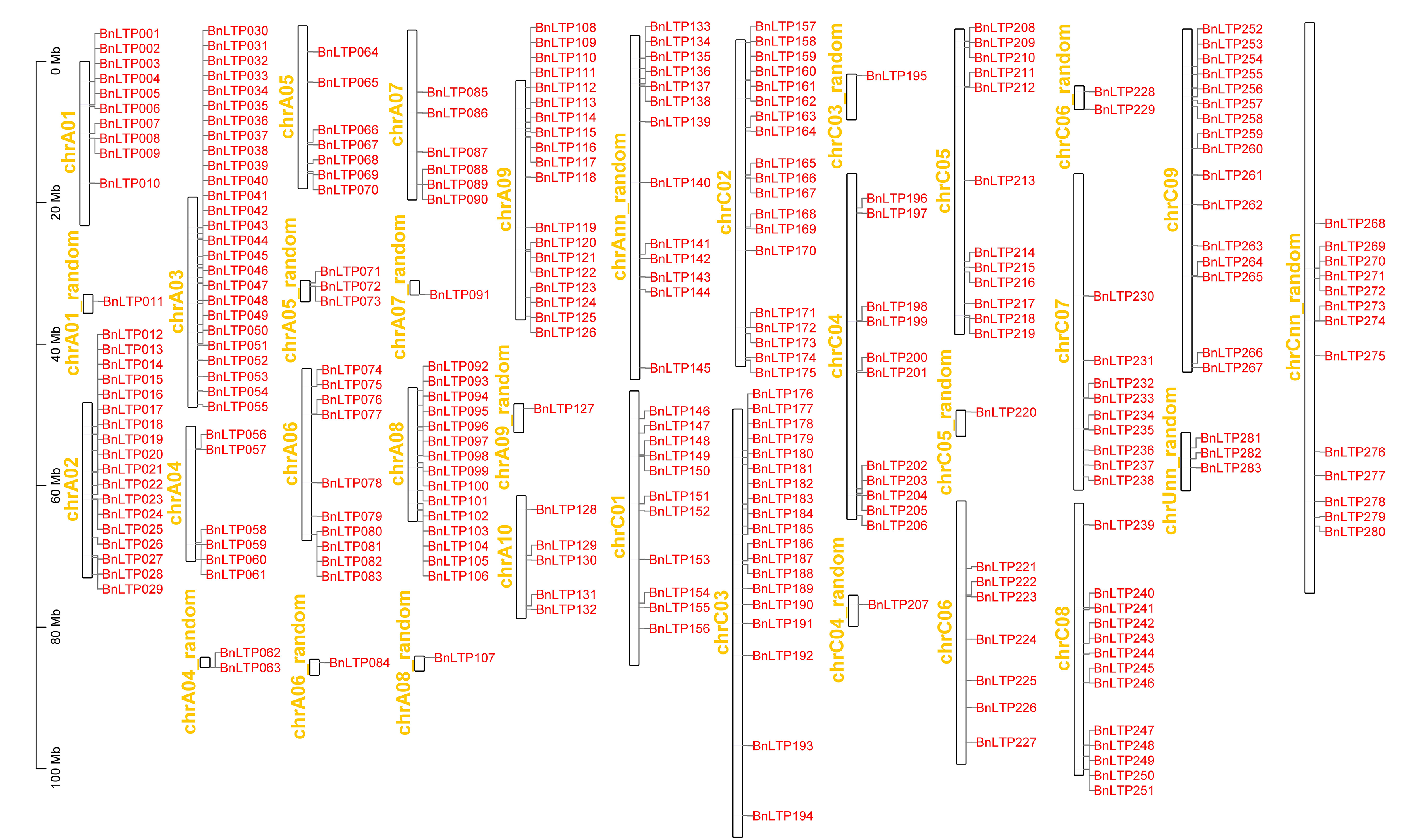

Supplement: Supplementary file 1 [file ijms-23-08372-s001.zip › ijms-1829603-supplementary/Figure S2.tif]

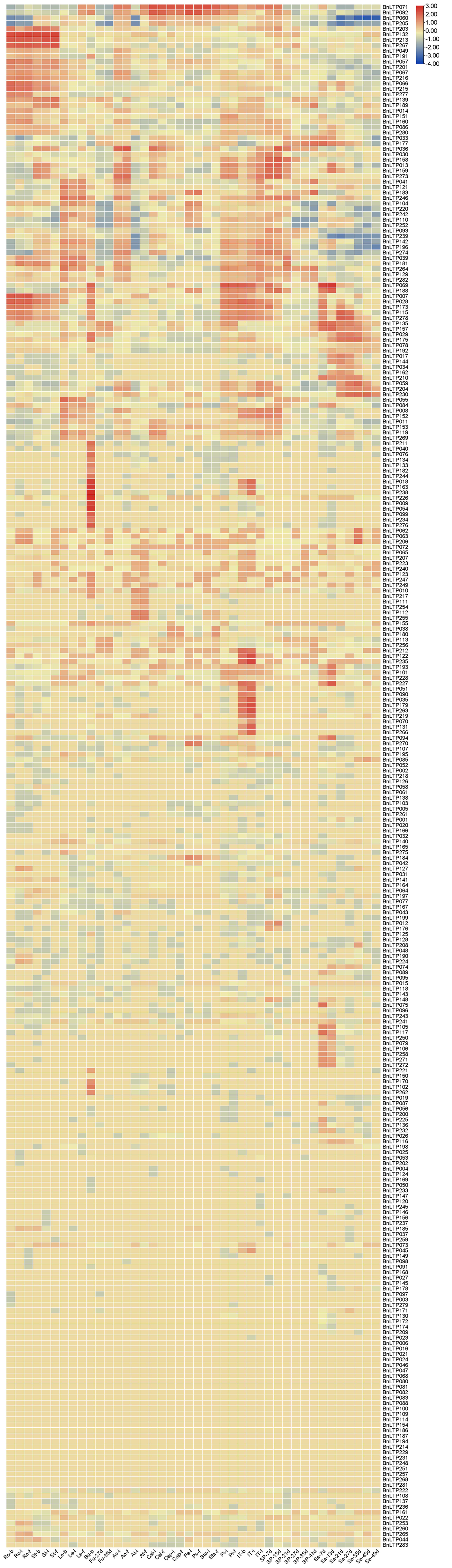

Supplement: Supplementary file 1 [file ijms-23-08372-s001.zip › ijms-1829603-supplementary/Figure S3.tif]

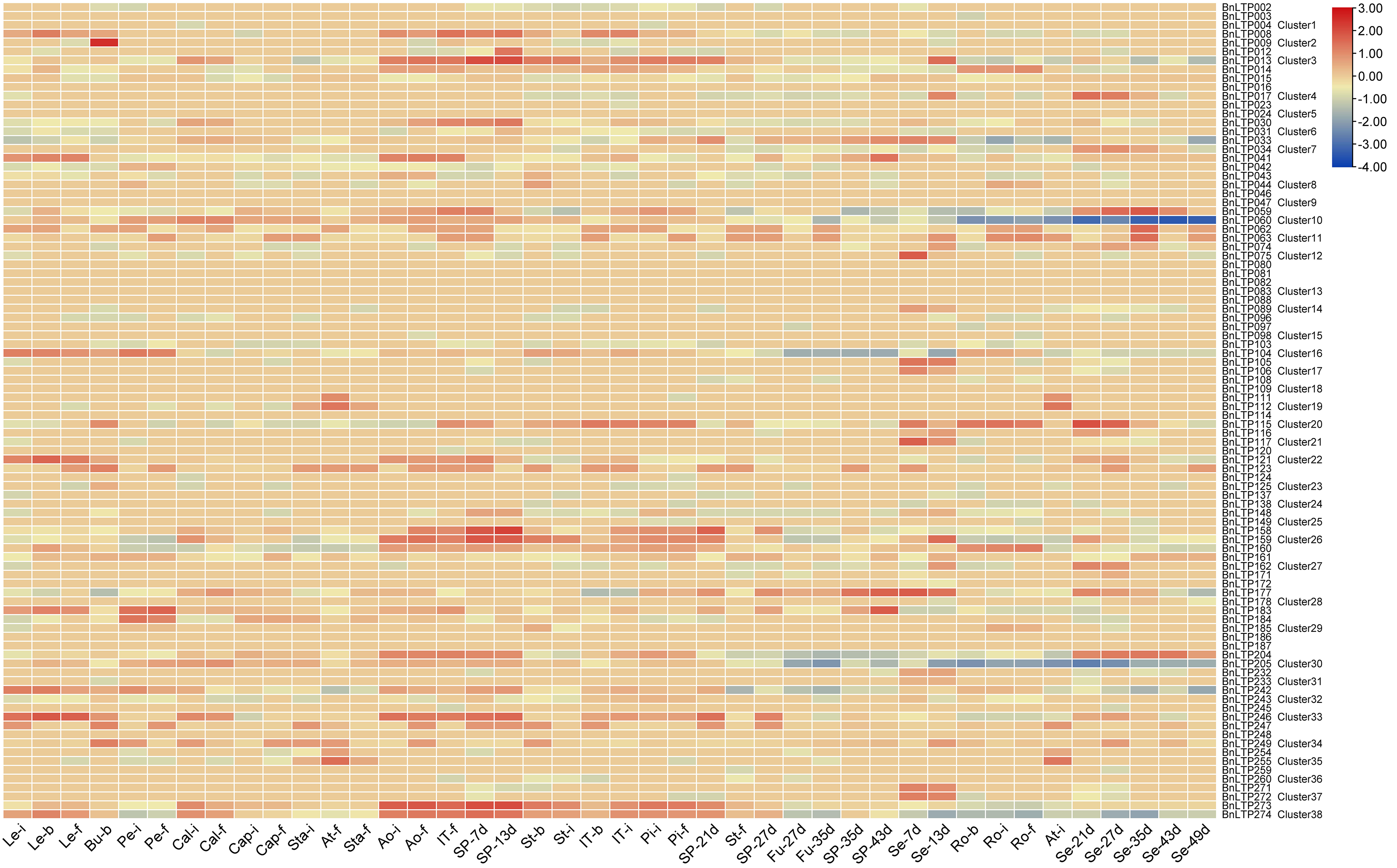

Supplement: Supplementary file 1 [file ijms-23-08372-s001.zip › ijms-1829603-supplementary/Figure S4.tif]

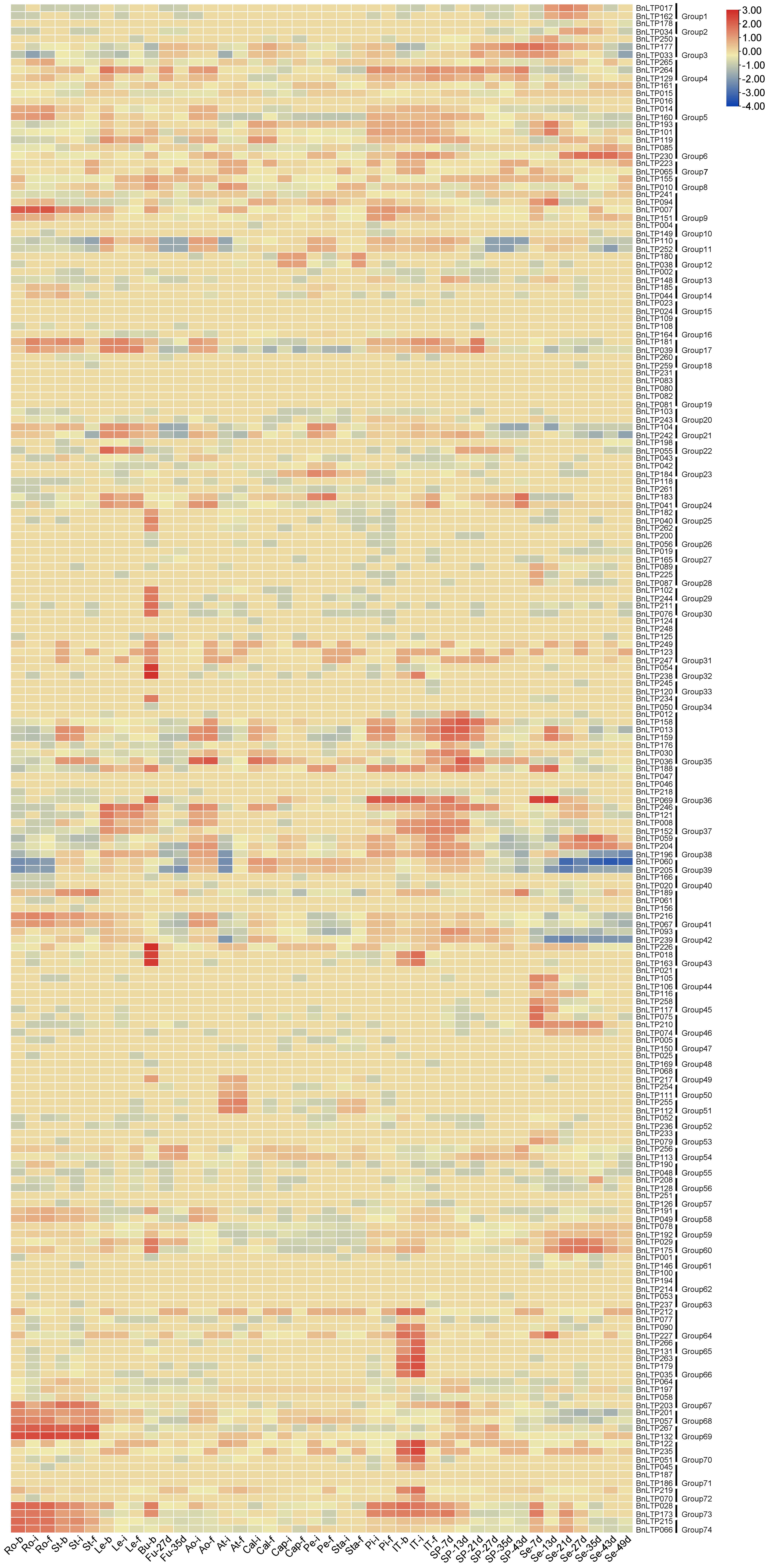

Supplement: Supplementary file 1 [file ijms-23-08372-s001.zip › ijms-1829603-supplementary/Figure S5.tif]

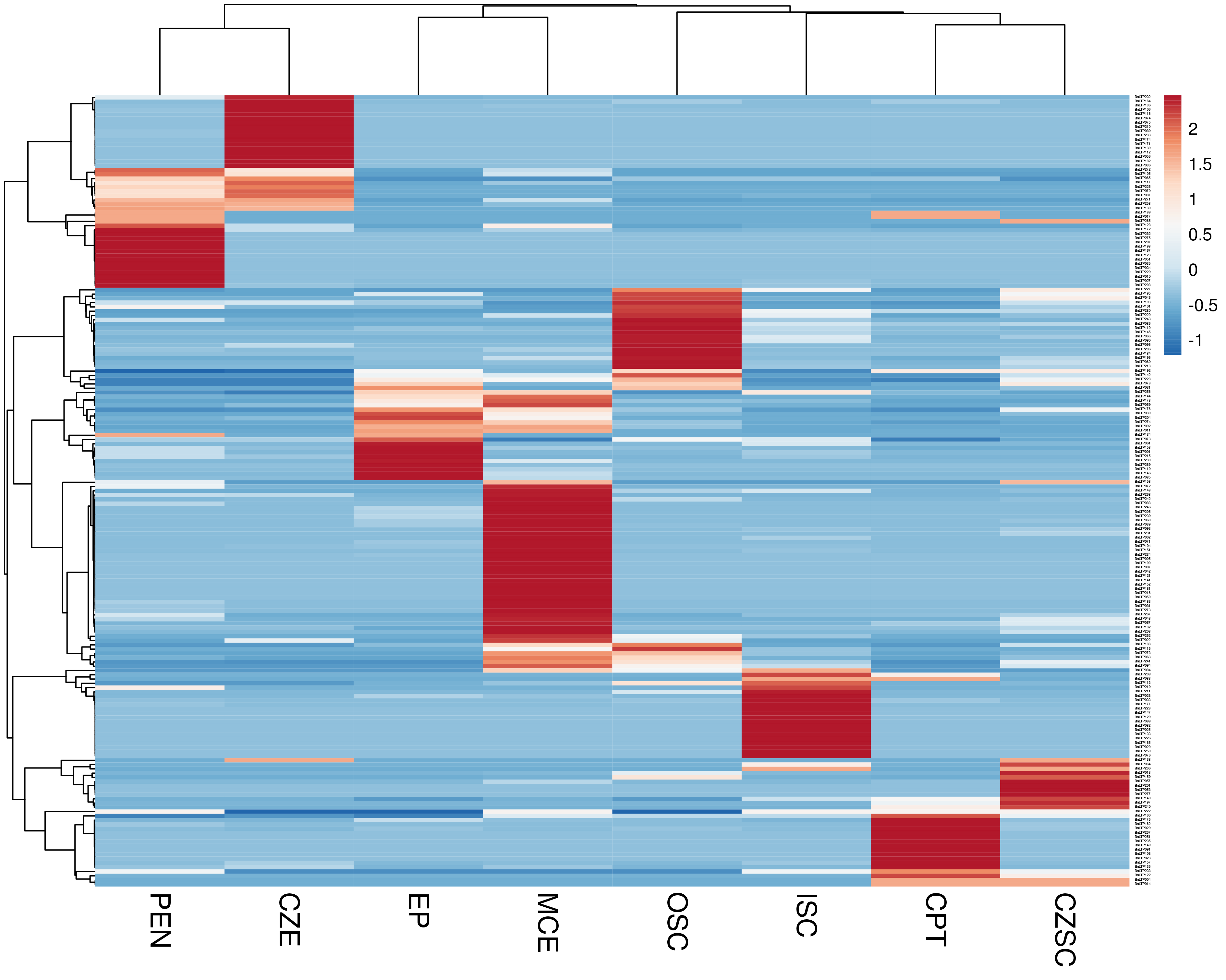

Supplement: Supplementary file 1 [file ijms-23-08372-s001.zip › ijms-1829603-supplementary/Figure S6.tif]

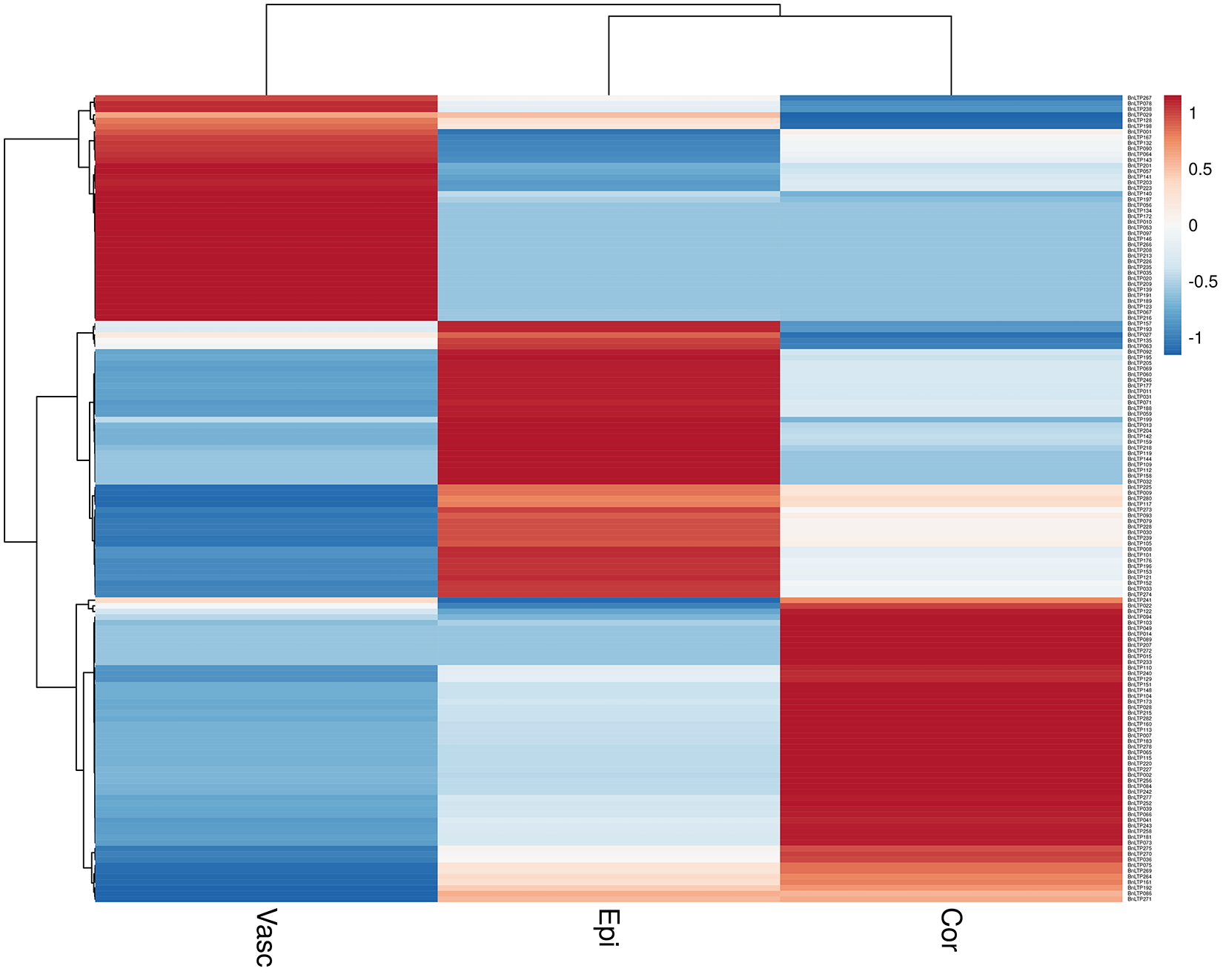

Supplement: Supplementary file 1 [file ijms-23-08372-s001.zip › ijms-1829603-supplementary/Figure S7.tif]

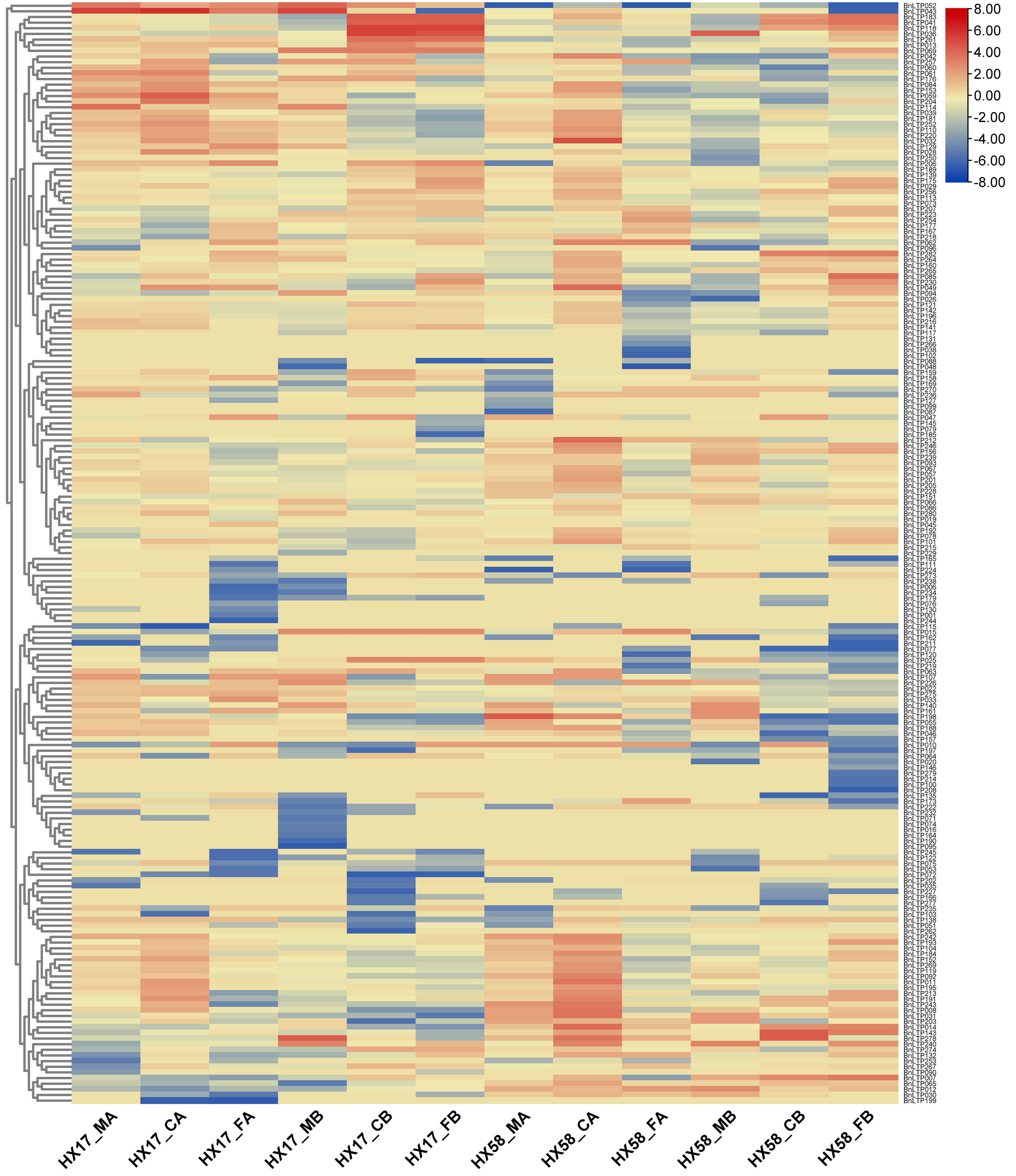

Supplement: Supplementary file 1 [file ijms-23-08372-s001.zip › ijms-1829603-supplementary/Figure S8.tif]
